# Urban Sanitation: New Terminology for Globally Relevant Solutions?

**DOI:** 10.1021/acs.est.3c04431

**Published:** 2023-10-11

**Authors:** Linda Strande, Barbara Evans, Marcos von Sperling, Jamie Bartram, Hidenori Harada, Anne Nakagiri, Viet-Anh Nguyen

**Affiliations:** † Eawag: Swiss Federal Institute of Aquatic Science and Technology, Department of Sanitation, Water and Solid Waste for Development (Sandec), Überlandstrasse 133, Dübendorf 8600, Switzerland; ‡ School of Civil Engineering, University of Leeds, Woodhouse Lane, Leeds LS2 9JT, U.K.; § Department of Sanitary and Environmental Engineering, Federal University of Minas Gerais, Av. Antônio Carlos 6627 - Campus Pampulha, Belo Horizonte 31270-901, Brazil; ∥ Graduate School of Asian and African Area Studies, Kyoto University, Yoshida-shimoadachi-cho 46, Sakyo, Kyoto 606-8501, Japan; ⊥ Department of Civil and Environmental Engineering, Kyambogo University, Kyambogo Road, Kampala, P.O. Box 1, Kyambogo, Uganda; # Institute of Environmental Science and Engineering (IESE), Hanoi University of Civil Engineering (HUCE), 55 Giai Phong Road, Hanoi 113068, Vietnam

**Keywords:** city-wide inclusive sanitation, fecal sludge, onsite, septic tank, pit latrine, sewer, sustainable development goals, wastewater

## Abstract

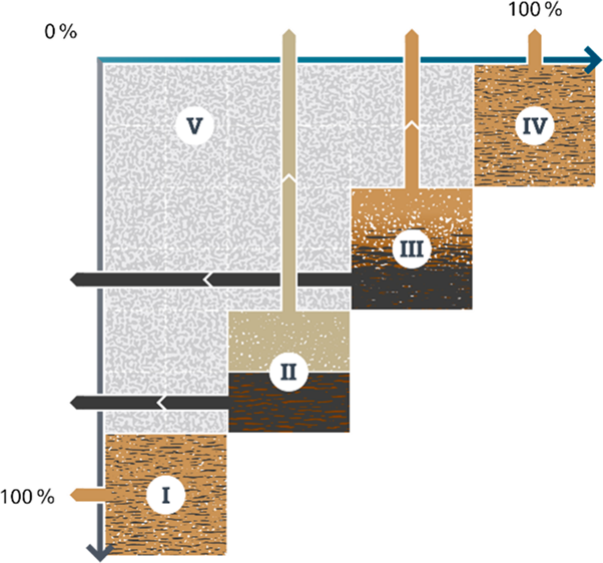

Progress toward Sustainable Development Goals for global access to safe sanitation is lagging significantly. In this Feature, we propose that misleading terminology leads to errors of categorization and hinders progress toward sanitation service provision in urban areas. Binary classifications such as “offsite/onsite” and “sewered/nonsewered” do not capture the need for “transport to treatment” or the complexity of urban sanitation and should be discarded. “Fecal sludge management” is used only in the development context of low- or middle-income countries, implying separate solutions for “poor” or “southern” contexts, which is unhelpful. Terminology alone does not solve problems, but rather than using outdated or “special” terminology, we argue that a robust terminology that is globally relevant across low-, middle-, and upper-income contexts is required to overcome increasingly unhelpful assumptions and stereotypes. The use of accurate, technically robust vocabulary and definitions can improve decisions about management and selection of treatment, promote a circular economy, provide a basis for evidence-based science and technology research, and lead to critical shifts and transformations to set policy goals around truly safely managed sanitation. In this Feature, the three current modes of sanitation are defined, examples of misconceptions based on existing terminology are presented, and a new terminology for collection and conveyance is proposed: (I) fully road transported, (II) source-separated mixed transport, (III) mixed transport, and (IV) fully pipe transported.

## Abstract 2

Urban sanitation requires a new vocabulary to overcome misconceptions and achieve progress on globally relevant solutions for safely managed sanitation.

## Introduction

Improvements in urban sanitation in the 19th and 20th centuries in Europe and North America resulted in significant inhibition of the spread of infectious disease. Subsequently, centralized waterborne sanitation was ranked as one of the top medical and engineering achievements of the 20th century.^1,2^ However, urban development and sewers have not always progressed hand in hand. In Harappa (modern day Pakistan), underground sewers for conveying human excreta were built as early as 3000 BC, with every house having a flush toilet.^3^ Since then, sanitation services have fluctuated with changes in civilizations, from the Cloaca Maxima in ancient Rome to no central sewer in London until the end of the 19th century.^2^ Diverse improvements have been made with flush toilets, piped sewers, and wastewater treatment, but this impetus has not been sufficient to solve the sanitation challenge on a worldwide basis. Currently, only 64% of urban residents globally are served by sewers,^4^ and it is not known how much wastewater actually receives effective treatment.^5^ The flush toilet is considered the gold standard as it conveniently removes feces and urine from sight and smell, but without adequate capture and conveyance, community-scale health and environmental benefits are obviously not achieved. This sanitation challenge in urban areas is increasing with rapidly growing cities (e.g., increase of 1.3 billion people between 2000 and 2017),^6^ along with climate change, water scarcity, and migration. Sustainable Development Goal (SDG) targets and sanitation as a human right are severely impeded by cultural reluctance, profound misconceptions, and honest ignorance, and achieving them will require us to overcome barriers that are preventing the roll out of appropriate solutions.

Research and practice efforts are often focused on advancing solutions at treatment facilities,^7^ but the protection of public health in urban areas relies as heavily on capture and conveyance to treatment, to avoid pathogen exposure at the source of production. The objective of this paper is to challenge common misconceptions around global paradigms for the management of sanitation, focusing on capture and conveyance to treatment. We postulate that these misconceptions lead to misinformed decisions that hamper progress in access to services in high-density urban areas and have profound detrimental downstream effects. We further argue that to derive globally relevant and sustainable solutions, we need a new terminology to overcome these misconceptions and provide a cogent basis for evidence-based science and technology research and contextualized programming.

## Current Framing of Sanitation Service Provision

**Figure 1:**
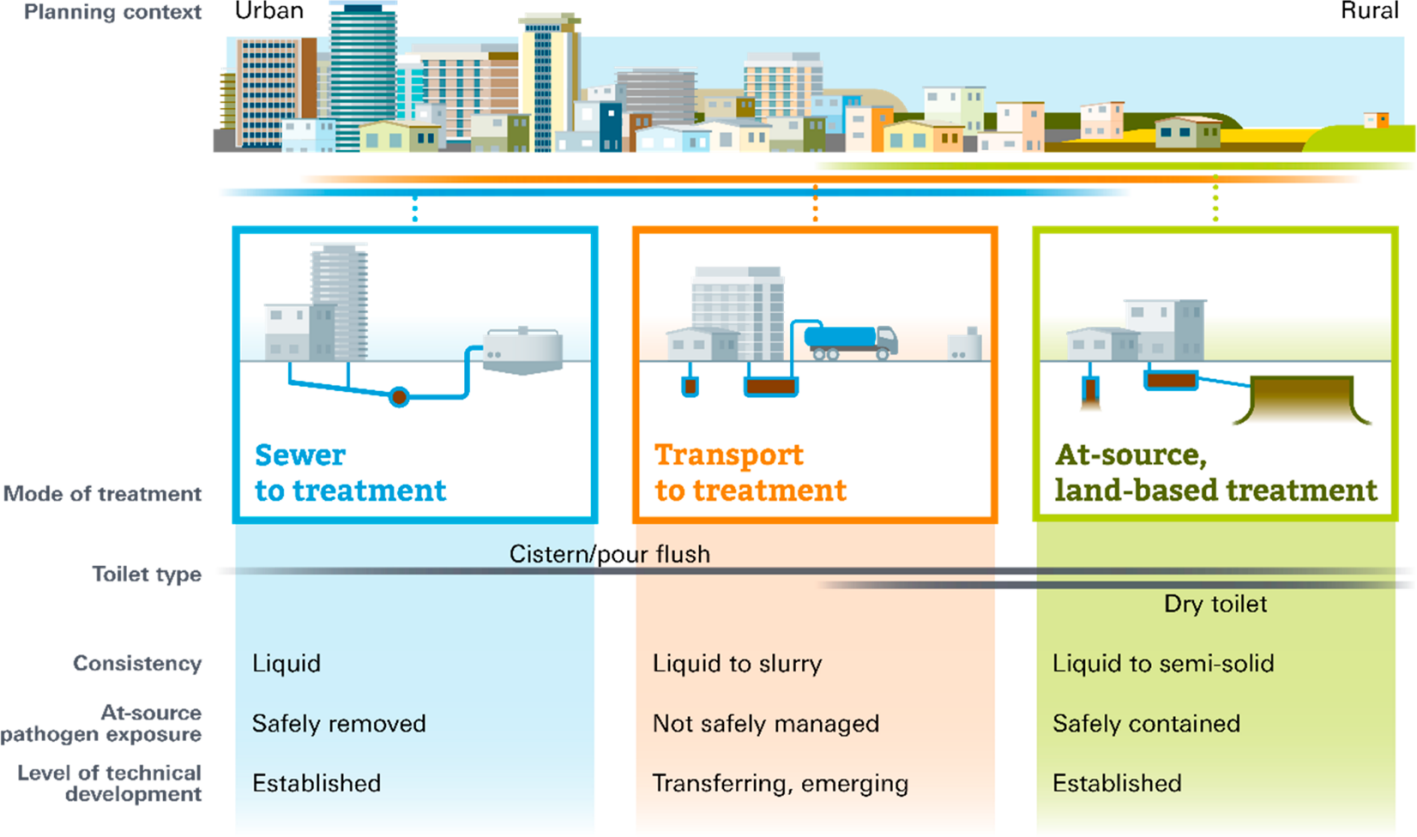
Three existing modes of sanitation service provision.

Global discourse around sanitation includes three broadly defined “modes” of service delivery (Figure 1), two of which are well-defined and one which is not: (1) sewer-based conveyance to treatment (also termed “offsite sanitation”), (2) at source containment followed by road-based conveyance to treatment, and (3) at source containment followed by land-based treatment. In a confusing and incorrect fashion, the latter two modes are often cited interchangeably as “onsite sanitation”.

Sewer-based systems designed for urban areas are mainly located where the population density is sufficient to justify their high capital costs. Globally, 70–95% of urban areas in upper-income countries are served by sewers, and 10–40% in lower- and middle-income countries.^4^ Sewer-based systems are designed to contain and convey municipal wastewater away from the population to a treatment facility and are also sometimes used for stormwater management.

In contrast, at source containment followed by land-based treatment sanitation options (commonly termed “onsite”) were developed for more sparsely populated areas in rural regions, or on the urban periphery. They rely on adequate land and environmental conditions for containment, followed by passive, land-based treatment within the soil close to or at the source of generation. These systems are used for all types of municipal wastewater or sometimes excreta and bathing water alone. Globally, they account for 58% of coverage in rural areas, ranging from 24% to 74%^4^ (but are not applicable in densely populated urban areas).

These two modes are entirely different from the mode of sanitation in urban areas that relies on at source containment and storage followed by road-based transport (central panel of Figure 1), sometimes termed “fecal sludge management”, or more recently “nonsewered sanitation”. This third mode relies on the capture of wastewater (i.e., blackwater with or without greywater) in various forms of at source containment (also commonly but not analogously termed “onsite”), with mechanical (trucks) or manual (carts) transport via a road network to treatment facilities. This mode is widespread in urban areas of low- and middle-income countries, where it accounts for 30–66% of coverage.^4^ This mode is diverse, ill-defined, and inadequately described by the terms “onsite”, “offsite”, or “nonsewered”.

We postulate that the binary classifications of “offsite/onsite” and “sewered/nonsewered” are misleading errors of categorization, and their use does not reflect the reality and leads to gross misunderstandings, for example, that “onsite” sanitation in urban areas is fulfilled with “simple technologies” or “simple solutions”, which are inexpensive and appropriate for low-income communities, and that these approaches provide levels of treatment equivalent to those associated with land-based treatment in rural areas. In reality, their management is much more complex and significantly less likely to result in the delivery of safely managed sanitation.^8,9^ In a similar fashion, “nonsewered” is problematic as it describes only what is not present and provides no information about the full complexity of sanitation in urban areas, such as how or whether wastewater is actually “transported to treatment”.

## How Did We Get Here?

Until the 1990s, “water and sanitation” projects in urban areas of low-income countries focused on drinking water provision. Sanitation was belatedly incorporated into the Millennium Development Goals (MDGs) in 2002. Great strides have been made in access to toilets, motivated by the acknowledgment that human dignity requires a place to safely and discretely relieve oneself of feces and urine. In 2010, 122 countries signed a U.N. resolution acknowledging the human right to safe drinking water and sanitation,^10^ and 78% of the world’s population now has access to at least a basic toilet.

“Pit latrines” and “septic tanks”, originally designed for rural areas, were transferred to fast growing and densifying urban contexts as “cheap” options to capture excreta or blackwater, without full consideration of what happens when they fill, leach, or overflow. These “simple” technologies were originally put in place as ad hoc or bridging measures until sewers could be constructed, but the sewers have never reached many city dwellers, be it due to 50-year planning cycles, slow construction, cost, or technical complexity. Around the world, sanitation solutions without sewers are considered temporary solutions. For example, in the United States where 25–30% of the population was served by nonsewered sanitation, the U.S. Environmental Protection Agency did not acknowledge that such approaches could be a sustainable option until 2003.^11^ This mindset of interim solutions and denial of their widespread application in low-, middle-, and upper-income countries has impeded appropriate management, research, and development of new or improved approaches.

Fortunately, this was acknowledged with the shift to a treatment focus in the SDGs, which has helped to improve the awareness of the importance of transport of wastewater to treatment. However, the result is still that a third of urban residents worldwide are served by neither sewer-based conveyance to treatment nor land-based treatment solutions, and this proportion is continually increasing. Between 2000 and 2020 in urban areas of India alone, 183 million people started using flush toilets connected to pits or tanks.^4^ An evaluation of sanitation in 39 cities found that half of the fecal waste in this form of sanitation leaks directly at source into the environment where people are living.^12^

## Why Does Terminology Matter?

The World Health Organisation categorizes sewer-based and rural land-based solutions as “established”,^13^ meaning that technologies are reliably understood to the level where globally accepted guidelines exist on how to design, build, and operate them. Research into activated sludge has been taking place for more than 100 years,^7^ and guidelines for land-based treatment with septic tanks^14^ and pit latrines^15^ have been in place for more than 70 years.

By contrast, solutions for full and safe management of sanitation in urban areas that rely on at source storage and road-based transport remain mostly as “emerging” or “transferring” solutions.^13^ This mode of service provision is widespread and rapidly gaining acknowledgment as a viable solution, although solutions have not yet been established. This is increasingly called “fecal sludge management” or “FSM” in the development sector, but the term is poorly defined.

An implicit problem with the term fecal sludge management is that it is used only in the context of sanitation in low- or middle-income countries, implying separate solutions for specific country contexts,^16^ or for the poorest people in urban areas of these contexts. This inherently colonialist construct contradicts the long-standing understanding that urban sanitation needs to be delivered as an entire system, providing appropriate services to everyone to generate the health, environmental, and social benefits for all and that sanitation systems need to safely separate humans from excreta.^17−20^ Classifying countries by income level is in itself problematic, as this simplification does not reflect reasons why countries are or remain “less developed” or “poor” (e.g., exploitation and colonialism).^16^ For lack of a better terminology, in this paper we are referring to low-, middle-, and upper-income countries.^16,21^ However, rather than using outdated, “special” terminology, we argue that a robust terminology that is globally relevant across low-, middle-, and upper-income contexts and that recognizes all legitimate (safe and sustainable) options in all contexts will advance safely managed sanitation and generate new solutions. We set out four examples of how confusing or ambiguous terminology hampers realistic assessments of the current situation and the planning of improvements.

### 1. Septic Tanks and Pit Latrines in Urban Areas Are in Fact Not Septic Tanks and Pit Latrines

The overall picture in urban areas without sewer systems is a chaotic mixture of inappropriately and haphazardly constructed solutions that attempt to contain and store wastewater onsite, with no level of standardization.^22^ Depending on the region, what are frequently termed “septic tanks” in urban areas actually range from permeable cess pits, through fully lined tanks with an overflow, to storage tanks with no outflow. They are of varying size, are clogged with solids, and often drain via overflow to open drains or nearby bodies of water.^23^ In conventional land-based treatment, a “septic tank” requires an engineered drain field for treatment, but adequate drain fields are not feasible in densely populated areas. Replacing the drain field with a “soakaway” does not provide adequate treatment to protect surface and groundwater.

Similarly, what are regularly termed “pit latrines” in urban areas range from soil pits to partially or fully lined storage, with highly heterogeneous layers of solids and liquids mixed with rubbish. In well-maintained, land-based treatment in rural areas, “pit latrine” waste is contained and disperses into the soil in a controlled manner. The management of such systems typically entails alternating two or more pits or to cover over a full pit and replace it by digging a new one. By contrast, in urban areas, with very different usage patterns, the filling rates are much higher and the pits require frequent emptying with transport to treatment, as there is no land available to dig a second pit when they become full. Wastewater that accumulates in onsite containment varies from 10 to >1000 L per person per day,^24,25^ with the amount that is contained and accumulates being much smaller than the total produced. Accumulation rates are so variable due to the range of onsite containment technologies, retention times, differences in household and commercial usage patterns, quality of construction, and collection practices.^24^

### 2. Existing Categories Are Not Analogous to “Safely Managed Sanitation”

Clearly, in urban areas having a so-called “septic tank” or “pit latrine” is not analogous to safely managed sanitation, as the liquid flows are not contained and are easily spread throughout the city.^23^ This is also exacerbated by flooding and washing out of containments.^24,26^ This partly stems from the misconception that onsite storage of wastewater is analogous to some form of treatment, whereas in fact, containments are for storing wastewater and are not designed for treatment. In land-based treatment, the removal of pathogens from septic tanks is achieved in the soil through the drain field and not in the septic tank. Furthermore, in practice, so-called “septic tanks” rarely settle out suspended solids, as they are not designed or maintained for the actual operating conditions.^27^ Assumptions about the safe containment of pathogens in onsite land-based treatment cannot be reliably transferred to dilute wastewater in urban areas.^15^ Furthermore, wastewater that is removed from containments (e.g., by vacuum truck) is frequently dumped into the environment, due to difficulties of transporting it via congested road networks, or due to a lack of viable alternatives.^28^ The result is that common pathways of exposure are open drains and market-bought produce and street food that are contaminated through washing.^29^

A further misconception is that sewer-based systems are analogous to safe and managed sanitation. In lower-income countries where sewerage coverage is low due to a lack of adequate funding and coordination, the majority of wastewater also remains untreated for the same reasons. In upper-income countries, treatment is never 100% and is more costly as it becomes more extensive.^30^ In areas with combined wastewater and stormwater management, combined sewer overflows of raw sewage during rainfall events translate to untreated excreta in recreational water, fish and shellfish harvesting areas, and agriculture.^31,32^ Increasing density and instability in weather patterns increase the likelihood of massive failures.^33^

### 3. Fecal Sludge Is Not a Useful Descriptor

The term “fecal sludge” conjures up images of feces (semisolid and low moisture content), and one could safely assume it was originally based on dry pit latrines in rural areas, where the accumulated contents would be relatively “dry”, “thick”, or sludge-like, if sludge is defined by consistency alone. However, it is not an accurate descriptor of what flows to onsite storage in dense urban areas of low- and middle-income countries. In this case, what is normally termed wastewater when it flows in a sewer is suddenly renamed as “fecal sludge”. This is misleading, because when wastewater includes water from pour- or cistern-flush toilets, in addition to cleansing, bathing, cooking, food waste, and rubbish, it is typically <5% total solids.^34,25^

It is also a misconception that “fecal sludge” is from only households, and global statistics on sanitation coverage are limited to households, schools, and healthcare facilities.^4^ However, a majority of people spend most of their waking time outside the household where they sleep at night, and in urban areas of low-income countries, a majority of meals are eaten on the street.^35^ Depending on the urban makeup, nonhousehold sources could be 50% of total wastewater, coming from offices, restaurants, markets, malls, small-scale manufacturing, and hotels.^36^ During “fecal sludge management” planning, these streams are rarely accounted for, resulting in inadequate management capacity.

### 4. Wastewater Streams from Human Feces Are Not All the Same, and This Has Significant Impacts on Treatment

Although the wastewater stored in containments is dilute, it has properties different from those of municipal wastewater arriving at treatment facilities through sewers. In addition to the high variability in containments, it is collected batch-wise individually, different fractions are emptied, and it is not homogenized during transport in a sewer.^24^ It can be similar or up to 2 orders of magnitude more concentrated in total solids, organic matter, and nutrients, with varying levels of stabilization. It is commonly thought that time since last emptied is equivalent to overall storage time and is a predictor of stabilization.^37^ However, stratification occurs with continual inputs of “fresh” excreta to containments, and storage is not analogous to anaerobic digestion that is process controlled at treatment facilities to optimize microbial degradation,^38^.^39^ Changes to wastewater during passive anaerobic storage have been observed to level off after 1 week,^40,41^ and average biochemical oxygen demand (BOD_5_) concentrations of almost 10 000 mg/L have been observed in the bottom regions of pit latrines.^42^

Wastewater in containment is also different from what is termed “sludges” in sewer to treatment systems. Fractions of wastewater in this case are not termed “sludge” until they have undergone additional processes.^36^ For example, primary sludge is settled and conventionally has a high level of total solids.^36^ Other sludges produced during treatment can be more dilute and are comprised mainly of microorganisms (biomass) from biological wastewater treatment. A treatment plant may include stages of sludge treatment, including thickening, digestion, dewatering, and hygienization, depending on the type of wastewater treatment process and the destination of the sludge.^43^ Scientific evidence about differences between blackwater that has been stored in containment in comparison to conventional wastewater sludges is mounting, including dewatering performance,^40,34,37,41,44^ protein-like fractions of extracellular polymeric substances (EPS),^40,41^ particle size distribution,^45,44^ fibers and lipids,^46,47^ rheological properties,^48,37^ and microbial communities.^49^

The assumption that waste streams have similar characteristics and properties has detrimental impacts on the design and operation of the conveyance and treatment. For example, dumping of stored wastewater in sewers can result in blockages due to higher than planned levels of inert solids. Co-treatment of wastewater without pretreatment (removal of solids) can cause serious operational problems ranging from incomplete removal of organics, cessation of nitrification, and filamentous growth to high sludge generation that can overload clarifiers.^50^

## Toward Terminology That Enables Globally Relevant Solutions for Urban Sanitation

Straightforward, clear, unambiguous, and globally relevant terminology, which captures how systems actually work, will help improve communication, understanding, and decision making. Fundamental to this is an improved terminology for modes of sanitation service provision to ensure wastewater arrives at treatment and treatment is appropriately designed. To achieve this, we propose a categorization specifically based on how excreta is safely contained and conveyed from the source of production, particularly the liquid flows of wastewater (combined and/or separate collection of blackwater, graywater, or urine).

**Figure 2:**
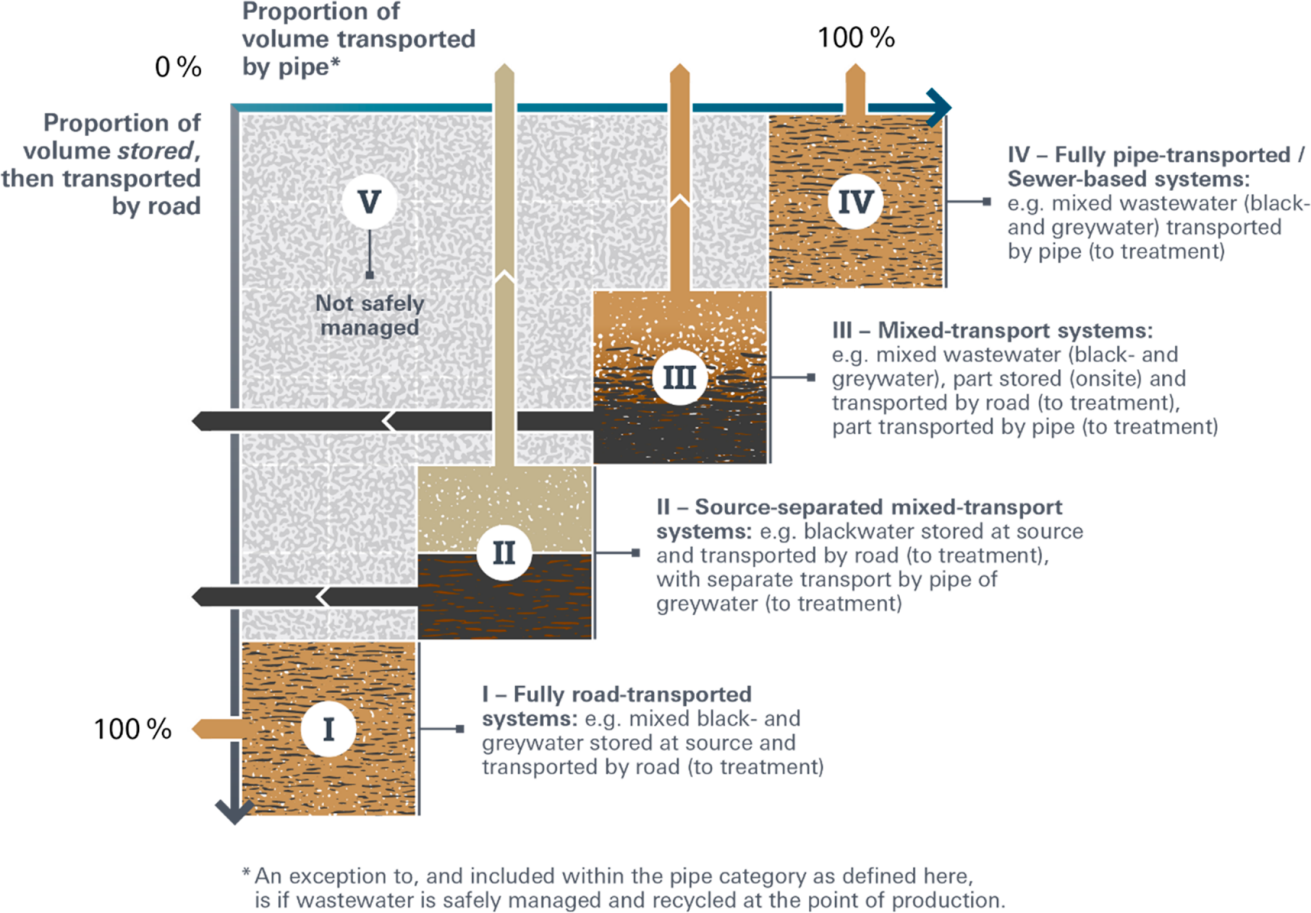
Categories of storage and transport from the point of production for wastewater (black- and graywater) in urban sanitation areas, based on the scale of individual buildings and/or the source of production. Categories I–IV have the potential to be “safely managed” provided all flows are delivered to adequate treatment, whereas category V is not “safely managed”.

We identify four broad categories of systems in urban areas with the potential to be “safely managed” by conveyance to functional treatment facilities. These are presented in the diagonal section of Figure 2. We assert that for wastewater to be safely managed in urban areas, if it is stored onsite, the containment should be impermeable (e.g., concrete or lined masonry), with either no overflow or an overflow going to a pipe network. All wastewater in urban areas needs to be transported away from the point of production to suitable treatment. We propose that there are two options for this, either via a pipe or by road. Pipe networks are defined here as fully enclosed and do not include open sewers. Road transport is defined as being performed with purpose-designed vehicles. An important exception that we have included within the pipe category as it is clearly distinct from road transport is if wastewater is safely managed and recycled at the point of production. The use of multiple categories is appropriate when planning for city-wide sanitation. Unsafely managed excreta, which is not safely contained and conveyed to treatment, falls in category V, the area above and to the left of the diagonal in Figure 2. Figure 2 represents flows from the point of production. In the 241 excreta flow diagrams (or SFDs) of city-wide sanitation on the SFD Promotion Initiative Web site,^51^ areas that are red or designated as not safely managed would fall in category V. The four categories of containment and conveyance for safely managed sanitation are further described in Table 1.

**Table 1:** Description and Examples of Categories of Storage and Transport for the Treatment of Wastewater (black- and graywater) in Urban Areas

categories	description	examples
(I) fully road transported systems: e.g., mixed black- and greywater stored at source and transported by road (to treatment)	Mixed black- and greywater are collected and fully contained in a sealed containment with no outlet. Independent of the number and frequency of users, the size of the containment is inversely proportional to the frequency of emptying and is relatively high.	• Large sealed tanks that collect gray- and blackwater and are emptied by contractors every 1–2 weeks include “Bayaras” at the household level in Egypt^52^ and the commercial level in Kampala^24^ and Dubai^53^ and community-scale ablution blocks in eThekwini.^54^
		• Onsite storage, including onsite treatment and water reclamation, includes Johkasou type treatment at the individual, smaller-scale building level in Japan^55^ or vermifiltration (e.g., Soubeyran housing cooperative in Switzerland).
(II) source-separated mixed transport systems: e.g., blackwater stored at source and transported by road (to treatment), with separate transport by pipe of greywater (to treatment)	Greywater is collected separately at the source of origin and may be treated and recycled onsite or piped to treatment via simplified sewers. Blackwater is collected in a sealed containment with no outlet. The size of the containment is inversely proportional to the frequency of emptying but likely smaller than those in category I.	• Separate greywater treatment takes place in many locations, including selected locations in Vietnam,^56^ San Francisco, USA, with >100 systems installed or in permitting,^57^ Helsinborg, Sweden, which has separate pipes for blackwater, greywater, and food waste collection (i.e., grinders and vacuum pipes), the town of Malmo, Sweden, which is testing three separate pipe networks for greywater, blackwater, and stormwater, and the Jenfelder AU development in Hamburg, Germany, with separate networks for blackwater and greywater with biogas production and water reclamation.^58^
(III) mixed transport systems: e.g., mixed wastewater (black- and greywater), part stored (onsite) and transported by road (to treatment), part transported by pipe (to treatment)	A mixture of black- and greywater from the household is collected in a sealed containment with an outlet connected to a pipe. The containment requires regular emptying of the settled solids.	• “Settled sewerage” has been reported in Zambia and Nigeria,^59^ where black- and greywater are collected in a relatively small interceptor tank or larger lined tank (commonly termed a “septic tank”), connected to pipes that are designed to carry a lower level of total solids than conventional sewers.
		• High-rise buildings in Tokyo and Osaka include treatment with produced sludge transported away by truck and treated wastewater partially reused on site and transported away by pipe with partial water reclamation for toilet flushing.^55^
(IV) fully pipe transported/sewer-based systems: e.g., mixed wastewater (black- and greywater) transported by pipe (to treatment)	Conveyance in sewers of gray- and blackwater transporting 100% of the volume to treatment.	• This approach is prevalent in 64% of urban areas globally (JMP). Sewers may be “conventional” or “simplified”.
		• “Simplified” sewers have smaller diameter pipes laid at shallower depths^60^ and have been implemented in Brazil,^61^ El Salvador, Pakistan, Tanzania,^62^ and Nairobi, Kenya.^63^

## Relationship between Storage and Emptying Frequency

**Figure 3:**
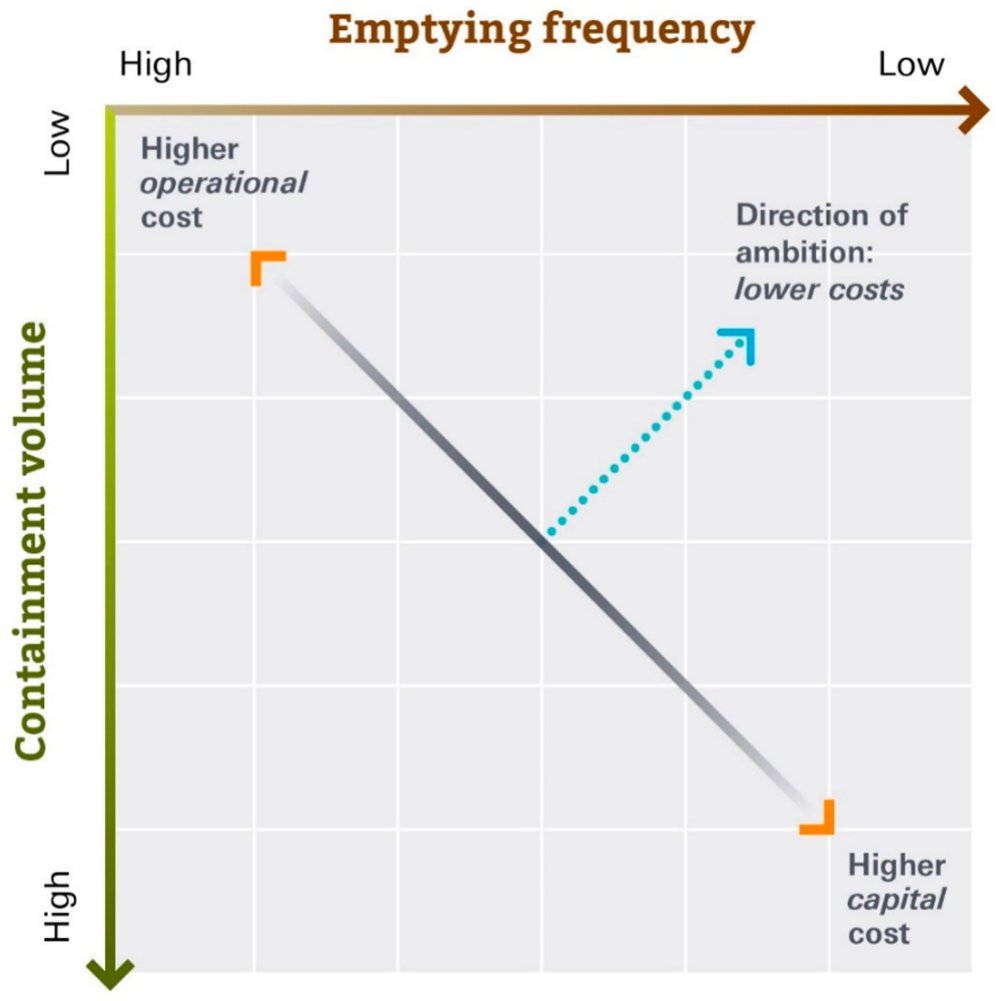
Comparison of volume and emptying frequency in relation to operational expenses (opex) and capital costs (capex) with “safely managed” conveyance to functional treatment facilities.

With containments in categories I–III, a trade-off between volume and emptying frequency is expected, which are good proxies for capital expenses (capex) and operational expenses (opex) (Figure 3). Large containments allow for longer periods between emptying but have higher capital costs and require more space. Improved approaches to separation of flows at source, storage, and management are needed to shift the overall trade-off to lower costs. An example of category II with smaller containments is a portable cartridge or container for blackwater that is collected frequently (<1 week) and replaced with a new cartridge (termed “container-based sanitation”) with separate management of greywater. “Container-based sanitation” has been implemented in Haiti, Kenya, Ghana, Peru, and Madagascar with no-flush toilets, and longer-term operating experience has demonstrated that it is cost-effective.^64^ Further innovations to reduce these costs could include in addition to blackwater, graywater, and urine, separate collection, treatment, and resource recovery from “light” graywater from bathing and “heavy” graywater and “greenwaste” from cooking.

## Implications for Management

Improved terminology allows for a more accurate assessment of the properties of flows arriving at treatment and hence improved treatment design. Moving toward fully sealed containments is also expected to reduce the huge variability in volumes and characteristics of flows resulting from current practices. This will allow engineers to develop appropriate processes with improved treatment efficiencies. For example, processes could be specifically selected to take advantage of the readily available organic matter in blackwater that has been stored for less than a week, such as anaerobic digestion, or conditioners could be used to improve dewatering of more stabilized blackwater, allowing for much smaller treatment footprints for dense, urban areas.^65^ Recent evidence on climate mitigation also suggests that blackwater should be moved as quickly as possible to treatment, where emissions can be controlled and captured.^39,66^

There is no specific hierarchy that ranks any of categories I–IV above another; however, different categories will be more relevant in specific contexts, and all present scope for further research, development, and innovation. Water is expensive and heavy to transport; the costs and energy required for pumping or transporting should be reduced as much as possible. Category II with separate management of greywater has the potential to reduce road-based transport and increase possibilities for nature-based solutions, including biological treatment such as vermifiltration.^67^ In addition, the separate collection of urine and feces also enables smaller containments and increased nutrient recovery and can even increase the capacity of existing infrastructure.^68,69^ Low- to no-flush technologies will greatly reduce total volumes of blackwater produced and dirty water that must be cleaned. Other research concepts include the “reinvent the toilet challenge”, which proposes no transport with mainly thermal or chemical treatment at source.^70,30^ These options also open up the possibility for community-scale systems.^71^ Smaller-scale, modular-based treatment technologies may also be more climate resilient in extreme weather events.^57^

Although the focus of the categories is on transport to treatment, it goes without saying that “established” treatment technology solutions themselves are no guarantee of safely managed sanitation and have to be coupled with adequate management. Safely managed sanitation requires adequate planning as a prerequisite for the functioning of any of the categories described above, together with active management of municipal solid waste (e.g., separation and transport by road) and stormwater (e.g., ditches, retention ponds, and gutters), which is a critical point where water, sanitation, and solid waste interact.^72^

## Call to Action

The world is not on track to meet the SDG target of universal access to safely managed sanitation; to achieve it, we need to overcome archaic assumptions and stereotypes about sanitation that are proving to be increasingly unhelpful. We recognize that terminology alone will not solve the problem, but we believe the current muddle and confusion, illogical binaries, and the vague use of terms impede critical shifts and transformations to set policy goals around truly safely managed sanitation. As laid out in this discourse, the terms “fecal sludge”, “fecal sludge management”, “nonsewered sanitation”, “onsite sanitation”, “off-site sanitation”, and misusage of “pit latrine” and “septic tank” have specifically led to a string of harmful misconceptions, especially for sanitation in urban areas. Instead, we propose the four categories for collection and conveyance: (I) fully road transported, (II) source-separated mixed transport, (III) mixed transport, and (IV) fully pipe transported. The use of the accurate and technically robust terminology proposed here will improve management decisions and the design of appropriate treatment facilities for the actual flows they are receiving and enhance possibilities for resource recovery and the circular economy. For example, sanitation service provision is often designed as “source-separated mixed transport systems” or “mixed transport systems”, but they are being managed as if they were “fully road transported systems”. This results in relatively small fractions of wastewater being safely delivered to treatment facilities, with a high risk to human health from exposure to pathogens at the source of generation. The same postulate applies to treatment facilities, where terminology from municipal wastewater treatment is often incorrectly applied to fecal sludge treatment facilities (e.g., drying beds following settling tanks as “secondary” treatment, or treatment of leachate as “tertiary” treatment), leading to misunderstandings of designed treatment objectives and inadequate protection of human and environmental health. We therefore call upon the sector to acknowledge a century of incremental compromise that is currently at the heart of ambiguities and to discard them in favor of terminology that will facilitate real transformation, effective accountability, and the delivery of urban sanitation for productive, healthy, and safe cities.
